# Assessing Energy Intake in Daily Life: Signal-Contingent Smartphone Application Versus Event-Contingent Paper and Pencil Estimated Diet Diary

**DOI:** 10.5334/pb.339

**Published:** 2016-12-20

**Authors:** Saskia Wouters, Viviane Thewissen, Mira Duif, Lilian Lechner, Nele Jacobs

**Affiliations:** 1 Open University of the Netherlands, NL

**Keywords:** Signal-contingent, Event-contingent, Between-meal snacking, Experience Sampling Method (ESM), Ecological Momentary Assessment (EMA), Estimated Diet Diary

## Abstract

**Objectives:**

Investigating between-meal snack intake and its associated determinants such as emotions and stress presents challenges because both vary from moment to moment throughout the day. A smartphone application (app), was developed to map momentary between-meal snack intake and its associated determinants in the context of daily life. The aim of this study was to compare energy intake reported with the signal-contingent app and reported with an event-contingent paper and pencil diet diary.

**Methods:**

In a counterbalanced, cross-sectional design, adults (N = 46) from the general population reported between-meal snack intake during four consecutive days with the app and four consecutive days with a paper and pencil diet diary. A 10-day interval was applied between the two reporting periods. Multilevel regression analyses were conducted to compare both instruments on reported momentary and daily energy intake from snacks.

**Results:**

Results showed no significant difference (B = 11.84, p = .14) in momentary energy intake from snacks between the two instruments. However, a significant difference (B = –105.89, p < .01) was found on energy intake from total daily snack consumption.

**Conclusions:**

As at momentary level both instruments were comparable in assessing energy intake, research purposes will largely determine the sampling procedure of choice. When momentary associations across time are the interest of study, a signal-contingent sampling procedure may be a suitable method. Since the compared instruments differed on two main features (i.e. the sampling procedure and the device used) it is difficult to disentangle which instrument was the most accurate in assessing daily energy intake.

## Introduction

In this study a signal-contingent smartphone app is compared with an event-contingent paper and pencil diet diary in assessing self-reported energy intake from between-meal snacks. It is important to assess the ability of innovative dietary assessment instruments to map snacking behavior in daily life. Indeed, mounting evidence shows that unhealthy food choices, especially between-meal snacks, contribute to the worldwide excess of energy intake and overweight (Piernas & Popkin, 2010; Nederkoorn, Houben, Hofmann, Roefs, & Jansen, 2010; Giesen, Havermans, Douven, Tekelenburg, & Jansen, 2010). Moreover, recent research suggests that determinants such as emotions and stress are crucial in predicting dietary behavior (Macht & Simons, 2010; O’Connor, Jones, Conner, McMillan, & Ferguson, 2008). In addition, between-meal snacking has been considered a suitable outlet for dealing with emotions and stress (O’Connor et al., 2008). Because snack intake as well as emotions and stress vary from moment to moment throughout the day, an instrument capable of mapping momentary between-meal snack consumption and its associated determinants in the context of everyday life, is required.

Several dietary assessment methods are considered suitable for gathering detailed information on daily dietary intake (Thompson & Subar, 2008). Estimated diet diaries and dietary recalls are used when detailed assessment of dietary intake during a relatively short, specified period, is required (Thompson & Subar, 2008). Estimated diet diaries, in which consumptions are reported concurrently, are generally used to gather dietary information over a 3 to 7 day period (Stephen, 2007). Dietary recalls encompass a 24 or 48 hour period and are usually completed by trained interviewers who ask respondents to recall what they have consumed during the previous day(s) (Stephen, 2007). Both assessment instruments have been developed exclusively for tallying and describing consumption. In addition, these methods have been adapted to include other information such as eating context (Mak et al., 2012; Matheson, Killen, Wang, Varady, & Robinson, 2004). However, in our opinion, the abovementioned dietary assessment instruments are less suitable to capture fluctuating determinants such as emotions. With the 24 or 48 hours recalls there seems to be risk of a recall bias. Robinson and Clore (2002) emphasized that people will report differently on their emotions depending on the time span between actual occurrence of experiences and retrieval from memory. In noncurrent reporting, when longer time frames are involved between occurrence and retrieval, people will rely on more global emotions, which are general in nature and not at all dependent on time or place (Tulving, 1984; Robinson & Clore, 2002). The estimated diet diary, however, seems a feasible instrument to assess emotions concurrent with snack events. Nevertheless, research has demonstrated that emotions are influenced by intake of consumptions (Desmet & Schifferstein, 2008; Macht & Dettmer, 2006) which may lead to a systematic bias in the reported emotions. Thus, neither the estimated diet diary nor the 24 or 48 hours recalls seem suited for this purpose.

A method, measuring at unpredictable random times during the day, is preferable for assessing determinants such as emotions and stress which are highly context-dependent and fluctuate throughout the day. These processes should be measured using a methodology that reflects the variety of emotions and eating occasions in daily life. Traditional questionnaires, developed for single measurements over a specific period, fall short in grasping these dynamic psychological processes of daily life. The Experience Sampling Method (ESM; Csikszentmihalyi, Hektner, & Schmidt, 2007; Csikszentmihalyi & Larson, 1987), a self-assessment diary technique also known as Ecological Momentary Assessment (EMA; Stone & Shiffman, 1994), is a suitable instrument to assess mental state and context in the course of daily life. ESM is an internationally used and validated research method, and has been successfully applied in both clinical and non-clinical populations (Jacobs et al., 2006; McKee, Ntoumanis, & Taylor, 2014; Myin-Germeys et al., 2009; Thewissen et al., 2011; Tournier, Sorbara, Gindre, Swendsen, & Verdoux, 2003). The strength of ESM lies in its ability to provide fine-grained, detailed pictures of human experience in natural settings (Scollon, Kim-Pietro, & Diener, 2003), and it is referred to as the gold standard for the measurement of emotions (Schwarz, Kahneman, & Xu, 2009). Snackimpuls, a smartphone app based on the Experience Sampling Method, was developed in order to gain insight into momentary between-meal snack intake and its associated determinants, such as emotions and stress, in daily life. Snackimpuls entails a signal-contingent protocol: the app emits multiple random audio signals (beeps) a day on several consecutive days, prompting participants to report current emotions, situational and social context, and between-meal snack intake since the previous beep.

The use of a signal-contingent methodology in assessing dietary intake, however, is still rather unexplored. Therefore, the aim of the present study is to compare moment-to-moment energy intake and total daily energy intake from between-meal snacks as measured by the Snackimpuls app, with the measurements of an estimated diet diary. It is hypothesized that both instruments are comparable in assessing (1) momentary, and (2) total daily energy intake from snacks.

## Methods

### Sample

**Figure 1:**
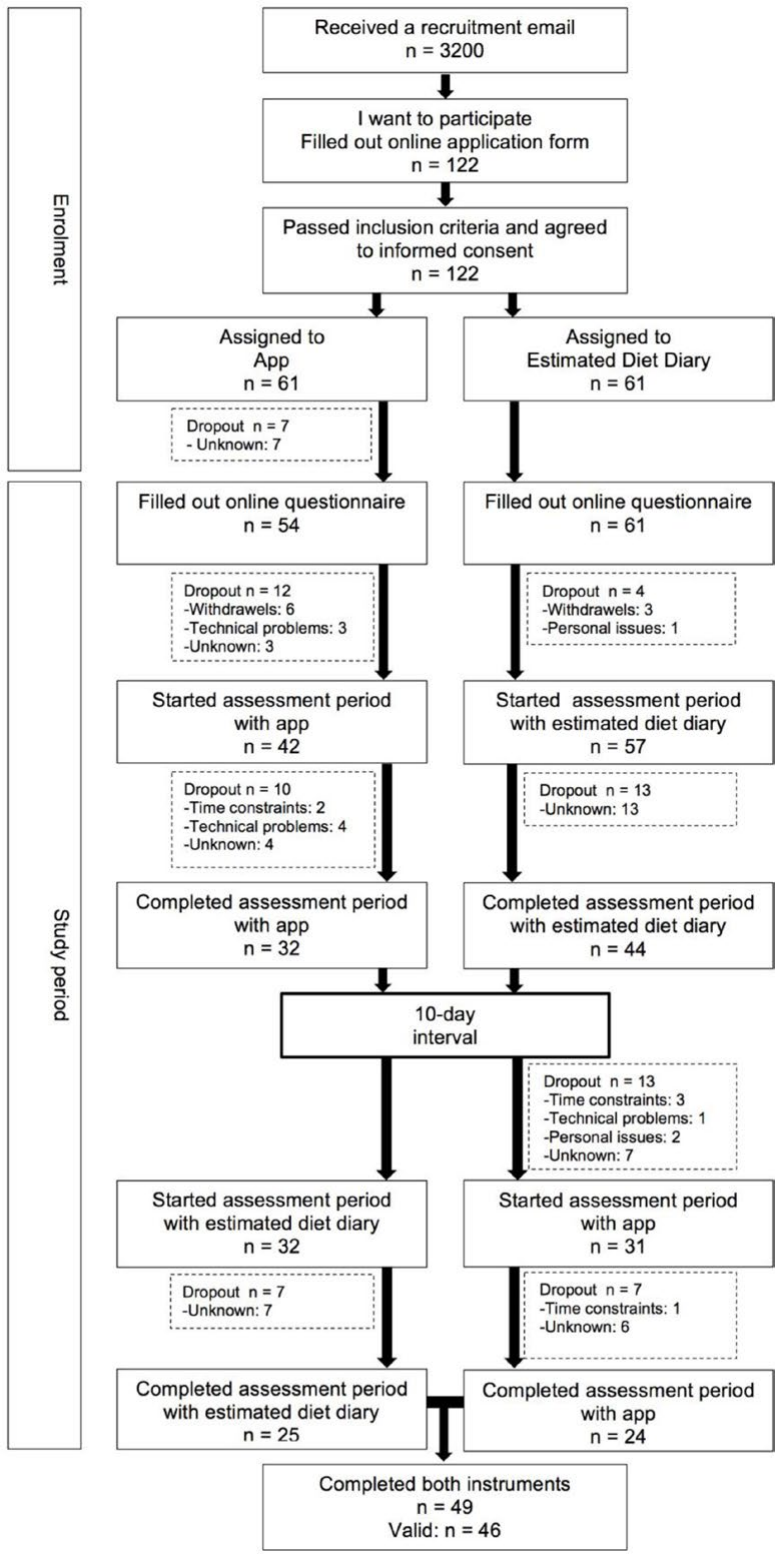
Flow chart participation.

All students enrolled in a propaedeutic course at the Open University of the Netherlands were approached by email. Students at this university are adults with heterogeneity in demographic variables such as previous education, age, marital status, employment status, income, and so forth. To participate, students had to be 20–50 years of age, as research has shown the largest increase in overweight individuals in recent years within this age group in the Netherlands (CBS Statline, 2014; Nationaal Kompas Volksgezondheid, 2014). Participants also had to be in possession of an Android smartphone. There were no criteria regarding Body Mass Index. In total, 122 students agreed to take part in the present study, of which 49 participants completed the study with both instruments (Figure 1). As a reward, participants received personal feedback based on their individual scores regarding eating behavior (Dutch Eating Behavior Questionnaire [DEBQ], van Strien, Frijters, Bergers, & Defares, 1986), daily activities, and affective states (Snackimpuls app). Moreover, participants had a chance of winning an Android tablet.

### Design and Instruments

A complete counterbalanced design with a 10-day interval between both instruments was applied to equal the distribution of respondent fatigue and carry-over effects across instruments (Figure 1). Participants were alternately allocated into one of two subgroups based on their starting instrument (Snackimpuls app or estimated dietary diary). The first assessment was conducted over four consecutive days from Wednesday to Saturday. To cover possible deviant snacking behavior during the weekend, one weekend day was included in each 4-day reporting period. After the 10-day interval, participants crossed over to the other dietary assessment instrument to complete their second 4-day assessment period, again from Wednesday to Saturday. The study was conducted during a normal week, excluding holidays, and participants were instructed to maintain their usual food intake during both assessment periods and to record all their consumptions and beverages outside of their main meals (i.e. breakfast, lunch, dinner) (Netherlands Nutrition Centre, 2011).

This study was approved by the Ethics Committee of the Open University of the Netherlands. In completing the online application form, participants agreed to an informed consent.

#### Online Questionnaire

Prior to the study participants received a short composite online questionnaire. Demographic variables such as age, weight, height, gender, marital status, and level of education were assessed. In addition, the Emotional Eating (13 items: α = .93), External Eating (10 items: α = .74), and Restraint Eating (10 items: α = .92) scales of the Dutch Eating Behavior Questionnaire ([DEBQ], van Strien et al., 1986) were administered to provide personal feedback on participants’ eating styles.

#### Snackimpuls Smartphone App

The Snackimpuls app was used to collect multiple daily assessments of between-meal snack intake, current emotions, and situational and social contexts over four consecutive days. Three days before starting with the Snackimpuls app, participants received an e-mail with user information (including instructions for downloading and installing the app), as well as a link to an instruction video on how to report snacks using the app. In addition, a training tool was integrated in the Snackimpuls app that provided participants with a single practice opportunity one day prior to their start with the instrument. During the assessment period, the Snackimpuls app produced 10 quasi-random audio signals (beeps) daily between 7:30 AM and 10:30 PM, prompting participants to report. In this study momentary energy intake was defined as energy intake from reported between-meal snacks between two beeps. With each reporting occurrence, the definition of a snack was presented in the first screen of the app as a reminder. Between-meal snacking was defined as all consumption of food and beverages, excluding main meals (i.e. breakfast, lunch, dinner) (Netherlands Nutrition Centre, 2011). Participants were asked: ‘Did you eat or drink anything between meals since the last beep?’ which could be replied with ‘Yes’ or ‘No’. If the anwer was affirmative, they were asked to report every product consumed and its quantity. The reporting time was automatically registered by the smartphone app. To help participants facilitate the recording of snack intake, the Snackimpuls app has a built-in search function. This search function consults a food composition table based on the scientifically accepted Dutch Food Composition Database (NEVO-online version 3.0, 2011, Rijksinstituut Volksgezondheid en Milieu). As the Dutch Food Composition Database does not contain natural units or household measures, the corresponding weights were derived from the database of The Netherlands Nutrition Centre (Caloriechecker, online version, 2013). Subsequently the weights of the household measures or naturel units (e.g. 1 cup of coffee contains 125 ml coffee) were converted into kilocalories in accordance with the Dutch Food Composition Database (NEVO-online version 3.0, 2011, Rijksinstituut Volksgezondheid en Milieu).

For each reported snack, participants could choose between two quantity options. Natural products, such as an apple, and products with standardized quantities, such as a Mars candy bar, could be reported either per piece or in grams (for solid foods) or milliliters (for fluids). Products with undetermined quantities such as yoghurt or tea, could be reported in relevant household measurements (i.e. a bowl or a cup) or in grams or milliliters. Participants could easily add products that were not already available in the search facility, using the keyboard of their smartphone. After completing the 4-day assessment period, participants were instructed to synchronize their data and uninstall the app. A pilot study has demonstrated the feasibility and usability of the Snackimpuls app (Wouters, Thewissen, Zamani, Lechner, & Jacobs, 2013).

The snack intake reported with the search facility of the app was automatically converted into kilocalories. This information was not visible to the participant. Any reported snacks that were not available in the search facility were converted into corresponding kilocalories by two researchers. The kilocalories for these products were extracted from the scientifically accepted Dutch Food Composition Database (NEVO-online version 3.0, 2011, Rijksinstituut Volksgezondheid en Milieu). If reported products were not available using the Dutch Food Composition Database, the food composition database of The Netherlands Nutrition Centre (Caloriechecker, online version, 2013) was consulted.

#### Estimated Diet Diary

The estimated diet diary was based on examples from literature (Thompson & Subar, 2008). Two days prior to their starting with this instrument, respondents received four paper booklets (one for each day) by regular mail. The first page of each booklet presented the definition of a snack. Instructions for completing the diary and examples were also included in each booklet. An event-contingent protocol was applied which required participants to report at every between-meal snacking occasion. More specifically, respondents were instructed to report every snack consumed and the time of consumption. In addition, respondents were encouraged to provide product information in as much detail as possible. Moreover, it was emphasized that quantities should be reported either in natural or standard units, household measures, or by grams or milliliters. After completing the 4-day period with this instrument, participants were asked to return the booklets in an addressed and stamped envelope. Using the same procedure as described above with the Snackimpuls app, two researchers converted the reported snacks in the estimated diet diaries into their corresponding kilocalories.

### Statistical Analyses

This comparison study focuses on energy intake at beep-level (momentary energy intake). The reported snack intake with the event-contingent estimated diet diary was clustered afterwards into the same time-frames as those of the app. Per participant, only days completed during both assessment periods were included in the analyses. When a participant missed a day (e.g. Thursday) in the reporting period with the estimated diet diary, the same day (i.e. Thursday) in the reporting period with the app, despite completion, was then excluded from the analyses and vice versa.

In ESM studies, participants are considered valid if they have reported at least 33% of the total number of beeps (Delespaul, 1995). Based on this criterion, only participants who replied to at least 14 beeps (of a total of 40 possible beeps) with the Snackimpuls app were included in the analyses. Participants who did not meet this criterion, were considered to be dropouts.

Because ESM data have a hierarchical structure with repeated momentary measurements (level 1), within each day (level 2), for each participant (level 3), multilevel linear techniques were used. Multilevel linear regression analyses were carried out using the xtmixed procedure in STATA/MP version 11 (Statacorp, 2009). Multilevel regression analyses were conducted to compare both instruments on momentary and daily energy intake. Due to the intense nature of experience sampling, the study involves a smaller sample size compared to cross-sectional survey studies, but the multiple assessments contribute to the method’s statistical power.

A mixed-design Anova analysis was applied to assess whether the order in which the dietary assessment instruments were used influenced the reported energy intake (differential transfer). Statistical analyses were conducted using STATA/MP (StataCorp., 2009). The level of significance for all analyses was defined at *p* < .05.

Dropout analyses were conducted (two-sample Wilcoxon rank-sum (Mann-Whitney) tests) to investigate significant differences in age, BMI, and eating styles (i.e. emotional, external, restraint) between participants who finished the study and the dropouts. In addition, Chi squared analyses were conducted to investigate significant differences in the distribution of gender, level of education, and starting instrument between these two groups.

## Results

Dropouts (*n* = 69) did not differ from the completers (*n* = 46) with regard to BMI (*n* = 77) (*Z* = –.19, *p* = .85), age (*n* = 115) (*Z* = 1.64, *p* = .10), emotional eating (*n* = 115) (*Z* = –.60, *p* = .55), restraint eating (*n* = 115) (*Z* = –1.14, *p* = .25), and external eating (*n* = 115) (*Z* = –.13, *p* = .90). Moreover, no significant differences were found in the distribution of gender (χ^2^ (1, *n* = 115) = .3.12, *p* = .08), level of education (χ^2^ (1, *n* = 115) = .58, *p* = .45), and starting instrument (χ^2^ (1, *n* = 115) = .1.68, *p* = .20) between both groups. Despite dropouts, the final sample of 46 adults was sufficiently counterbalanced: 24 adults started with the app and 22 started with the estimated diet diary. Mean age of participants^1^ (37 females (80%), 9 males (20%)) was 35.2 years (SD = 8.5, range 21–50 years), mean BMI was 23.5 (SD = 3.27, range 16.7–33.5) and 35 of the participants (76.1%) had a higher vocational or academic degree. In total, 177 days per instrument were included in the analyses (40 participants completed 4 days per instrument, 5 participants completed 3 days per instrument, and 1 participant completed 2 days per instrument).

Study participants yielded 1272 answered beeps (69.13% of the maximum number of beeps) with the Snackimpuls app. This is consistent with compliance rates in previous ESM studies in similar samples (Silvia, Kwapil, Eddington, & Brown, 2013). Of the total number of answered beeps, in 710 occasions participants indicated that they did consume between-meal snacks (Table 1). No snack intake was reported at 562 beeps. On group level, between-meal snacking (Table 2) resulted in a mean momentary energy intake per respondent of 137 kcal (SD = 52) and a mean daily energy intake of 529 kcal (SD = 217).

**Table 1:** Descriptives of momentary reports in which snacks were reported (N = 46).

	*Momentary reports in which snacks were reported*			
Instrument	Total number of beeps in which snacks were reported	% of max. number of beeps*	M Mean number of beeps in which snacks were reported*	Range of beeps in which snacks were reported

*Snackimpuls app*	710	38.6	15.4	4 – 28
*Estimated Diet Diary*	934	50.8	20.3	10 – 30

* the reported snack intake of the estimated diet diary was clustered into the same time frames as the app.

**Table 2:** Descriptives of energy intake (kcal) from between-meal snacking (N = 46).

	Momentary energy intake from snacks*		Daily energy intake from snacks*	
Instrument	M ± SD	Range	M ± SD	Range

*Snackimpuls app*	137 ± 52	46 – 281	529 ± 217	101 – 896
*Estimated Diet Diary*	123 ± 55	16 – 292	639 ± 301	70 – 1356

* means were calculated for each participant to obtain group means.

Based on the allocation to the corresponding beeps of the app time schedule, the clustered reported snack intake of the estimated diet diary yielded 934 beeps (50.8%) with snack intake (Table 1). On group level, between-meal snacking (Table 2) resulted in a mean momentary energy intake per respondent of 123 kcal (SD = 55) and a mean daily energy intake of 639 kcal (SD = 301).

The mixed design Anova showed no differential transfer (F(1,44) = 3.29, *p* = .08), indicating that the sequence of administration of instruments had no effect on reported energy intake.

Results of the multilevel regression analyses showed no significant difference in momentary energy intake between the two instruments (B = 11.84, SE = 8.03, *p* = .14). Results demonstrated that the Snackimpuls app is comparable with the estimated diet diary in assessing energy intake at beep-level. However, a significant difference between the two instruments was found with regard to energy intake on a daily basis (B = –105.89, SE = 37.19, *p* < .01). Reported daily energy intake was significantly higher with the estimated diet diary than with the Snackimpuls app. This indicates that the Snackimpuls app is not comparable with the estimated diet diary in assessing daily energy intake. A further in-depth analysis showed a significant difference (t(45) = 6.79, *p* < .01) between the app (M = 15.43; SD = 5.44) and the estimated diet diary (M = 20.30; SD = 6.62) with regard to the mean number of beeps in which snacks were reported during the research period.

## Discussion

The aim of this study was to compare energy intake reported with a signal-contingent smartphone app versus an event-contingent paper and pencil estimated diet diary. Results showed that both instruments were comparable on reported momentary energy intake. However, our findings also demonstrated that significant more daily energy intake was reported with the event-contingent paper and pencil diet diary. The in-depth analysis showed that the signal-contingent app yielded significantly fewer mean momentary snack reports compared with the paper and pencil diet diary. Apparently, the number of momentary assessments in which snacks were reported seems to play a role in the presently obtained discrepancy at daily level. This might be due to the fact that in the current study the instruments differed on two main features: the sampling procedure (signal-contingent versus event-contingent) and the device used (smartphone versus paper and pencil). Signal-contingent reporting, where participants are prompted to report by a signal of their smartphone, tends to be more intrusive than event-contingent reporting, where participants report on every between-meal snacking occasion. Time-signals may interrupt ongoing activity, whereas event-contingent reporting is provided as the event occurs (Reis, Gable, & Maniaci, 2014). Moreover, the largest source of missing data in time-sampling research, by far, is non-response or failure to respond to the daily life questionnaires (Silvia et al., 2013). Although compliance in the present study was comparable with previous research based on the experience sampling method (Silvia et al., 2013), it is conceivable that dietary reporting based on a signal-contingent protocol is more demanding than with an event-contingent protocol, resulting in fewer observations.

In addition, the type of device used (i.e smartphone versus paper and pencil) might have contributed to the presently obtained discrepancy. A paper and pencil method lends itself for back- and forward-filling to compensate for missed events or to anticipate for coming events. Smartphone technology data, however, are time stamped, which has the advantage of providing more insight into compliance regardless of the sampling procedure (signal-contingent or event-contingent). It is conceivable that back- and forwardfilling with the paper and pencil diet diary in the current study might have contributed to more observations compared to the signal contingent smartphone app.

Research has shown that electronic reporting on dietary intake was experienced as more acceptable than traditional paper and pencil methods (Illner et al., 2012). The question arises whether a blended protocol, within smartphone technology, might solve the pros and cons of both sampling procedures. If the signal-contingent protocol of the app (used for measuring determinants such as emotions), were extended with an event-contingent protocol to measure energy-intake, it seems plausible that the comparability with the estimated diet diary in terms of reported energy intake would increase. Combining a signal-contingent protocol with an event-contingent protocol, however, may have its weaknesses as well. Research has already demonstrated that the demands of frequent event-contingent food recording may discourage respondents from participating and cause others to drop out (Thompson, Subar, Loria, Reedy, & Baranowski, 2010). Moreover, with signal-contingent reporting the respondent’s burden is considered high as a result of the repeated sampling in the natural environment and the intrusiveness of the signal (Hufford, 2007). Hence, it is conceivable that respondent’s burden will increase when both protocols are combined. Additionally, even when a smartphone is used, event-contingent recording still enables back- and forward-filling, leading to incorrect time stamps. Thus, in our view the advantages of a blended protocol may not outweigh the potential threat of increased burden and invalid data. More research towards such a blended protocol is needed in order to further clarify its strengths and weaknesses.

Our study showed similarities and discrepancies between a signal-contingent smartphone and an event-contingent paper and pencil method in assessing energy intake from snacks. It has been pointed out that no dietary assessment instrument based on self-reports can measure dietary intake totally free of error whether using conventional formats such as paper and pencil, or innovative technologies such as smartphones (Freedman, Schatzkin, Midthune, & Kipnis, 2011). Our study demonstrates the comparability of a signal-contingent app with an event-contingent paper and pencil diet diary in assessing momentary energy intake. The instrument of choice will ultimately depend on the research purpose. A signal-contingent sampling procedure, using smartphone technology, seems preferable when momentary associations across time are the interest of study. Future research with the signal-contingent app can contribute to a better understanding of momentary energy intake from between-meal snacks and its associated fluctuating determinants in daily life. As between-meal snacking has increased over the past decades contributing to energy intake (Piernas & Popkin, 2010; Nederkoorn et al., 2010; Giesen et al., 2010) knowledge and understanding of snacking behavior is indispensable in modern health research.

The study’s main limitation pertains to the generalizability of the results because the current sample was small, predominantly female, and highly educated. However, despite the study’s relatively small sample size (N = 46), statistical power to detect differences at beep-level was preserved, as participants provided multiple assessments a day during consecutive days. In addition, the dropout rate in this study is notable. Reported reasons for dropping out were primarily related to time constraints, technical issues related to an Android update, and personal cirsumstances such as sudden change of holiday plans. Analyses showed a non-systematic dropout with regard to participant’s BMI, age, eating styles, gender, level of education, and starting instrument. As respondents initially received no personal benefit for participating in this study, incentives in the form of personal feedback and the chance of winning a tablet were added. Although previous research has demonstrated the demanding nature of food recording (Thompson et al., 2010) the dropout rate (60%) in the current study was still high. However, it should be noted that 31% (n = 36) of the participants (N = 115) dropped out either before starting with the assigned dietary assessment instrument (n = 23), or during the 10-day interval (n = 13). This might be due to the low compensation that participants received for their participation combined with the workload imposed by consecutively completing two different dietary assessment instruments. Indeed, a follow-up study (N = 382) with the same incentives, solely using the app during 7 consecutive days, showed a significant lower attrition rate (30%; n = 113) (Wouters, Jacobs, Duif, Lechner & Thewissen, submitted). The high number of dropouts in this comparison study might have influenced the results. It seems plausible that particularly individuals with high perseverance or interest in nutrition, did finish the study. This adds to the limations with regard to the generalizability of the results. Another limitation concerns a potential risk of underreporting with the app. The app was programmed in such a way that participants only received a follow-up question to report their snack intake in case they indicated that they had consumed a between-meal snack since the previous beep. It cannot be excluded that this kind of sampling might have encouraged individuals to choose the ‘no’ option, indicating that they did not consume any between-meal snacks. Although it has been demonstrated that missing data in ESM research are typically the result of individuals entirely ignoring a signal (causing all items to be missing for that beep), partial response may occasionally occur (Silvia et al., 2013). In case of conditional questions, future research should consider including filler questions in order to ensure that choosing ‘no’ would not abbreviate the questionnaire. A final limitation concerns the accuracy of reported snacks. In the app a search function was included to facilitate detailed reporting of between-meal snacks. However, no detailed list was provided when completing the estimated diet diary. As the support for detailed reporting differed between the instruments, it can not be excluded that reportings with the estimated diet diary were less precise.

## Conclusions

Although the signal-contingent app is comparable with an event-contingent paper and pencil diet diary in assessing momentary energy intake, both instruments differ in capturing total daily snack consumption. On daily basis, significantly more energy intake was reported with the event-contingent paper and pencil diet diary. As the compared instruments differed on two main features (i.e. the sampling procedure and the device used) it is difficult to disentangle which instrument was the most accurate in assessing daily energy intake. As at momentary level both instruments were comparable in assessing energy intake, research purposes will largely determine the sampling procedure of choice. When momentary associations across time are the interest of study, a signal-contingent sampling procedure, using a smartphone device to obviate the risk of back- and forward filling, may be a suitable method. Signal-contingent smartphone apps can provide researchers, clinicians, and dietitians insight into momentary between-meal snacking and the associated determinants, which may be helpful in preventing and addressing unhealthy snacking behavior.

Additional information regarding Snackimpuls can be found on its website (www.snackimpuls.ou.nl).

## Footnotes

Students at the Open University are adults with heterogeneity in demographic variables such as previous education, age, marital status, employment status, income, and so forth. The mean age of students that pass their propaedeutic exam is 39 years.

## References

[1] CBS Statline (2014). Leefstijl, preventief onderzoek: persoonskenmerken. http://statline.cbs.nl/StatWeb/publication/default.aspx?VW=T&DM=SLNL&PA=81177NED&D1=38-42&D2=0-12%2c33-37&D3=0&D4=l&HD=120626-1659&HDR=T&STB=G1%2cG2%2cG3.

[2] Csikszentmihalyi M., Hektner J. M., Schmidt J. A. (2007). Experience Sampling Method, measuring the quality of everyday life.

[3] Csikszentmihalyi M., Larson R. (1987). Validity and reliability of the Experience-Sampling Method. Journal of Nervous and Mental Disease.

[4] Delespaul P. (1995). Assessing Schizophrenia in daily life: The experience sampling method.

[5] Desmet P. M. A., Schifferstein H. N. J. (2008). Sources of positive and negative emotions in food experience. Appetite.

[6] Freedman L. S., Schatzkin A., Midthune D., Kipnis V. (2011). Dealing with Dietary Measurement Error in Nutritional Cohort Studies. Journal of the National Cancer Institute.

[7] Giesen J. C. A. H., Havermans R. C., Douven A., Tekelenburg M., Jansen A. (2010). Will Work for Snack Food: The association of BMI and Snack Reinforcement. Obesity.

[8] Hufford M. R., Stone A. A., Shiffman S., Atienza A. A., Nebeling L. (2007). Special methodological challenges and opportunities in ecological momentary assessment. The Science of Real Time Data Capture: Self-Reports in Health Research.

[9] Illner A-K., Freisling H., Boeing H., Huybrechts I., Crispim S. P., Slimani N. (2012). Review and evaluation of innovative technologies for measuring diet in nutritional epidemiology. International Journal of Epidemiology.

[10] Jacobs N., Rijsdijk F., Derom C., Vlietinck R., Delespaul P., van Os J., Myin-Germeys I. (2006). Genes making one feel blue in the flow of daily life: A momentary assessment study of gene-stress interaction. Psychosomatic Medicine.

[11] Macht M., Dettmer D. (2006). Everyday mood and emotions after eating a chocolate bar or an apple. Appetite.

[12] Macht M., Simons G., Nyklíček I., Vingerhoets A., Zeelenberg M. (2010). Emotional eating. Emotion regulation and well-being.

[13] Mak T. N., Prynne C. J., Cole D., Fitt E., Roberts C., Bates B., Stephen A. M. (2012). Assessing eating context and fruit and vegetable consumption in children: new methods using food diaries in the UK National Diet and Nutrition Survey Rolling Programme. International Journal of Behavioral Nutrition and Physical Activity.

[14] Matheson D. M., Killen J. D., Wang Y., Varady A., Robinson T. N. (2004). Children’s food consumption during television viewing. The American Journal of Clinical Nutrition.

[15] McKee H. C., Ntoumanis N., Taylor I. M. (2014). An ecological momentary assessment of lapse occurrences in dieters. Annals of Behavioral Medicine.

[16] Myin-Germeys I., Oorschot M., Collip D., Lataster J., Delespaul P., van Os J. (2009). Experience sampling research in psychopathology: Opening the black box of daily life. Psychological Medicine.

[17] Nationaal Kompas Volksgezondheid (2014). http://www.nationaalkompas.nl/gezondheidsdeterminanten/persoonsgebonden/overgewicht/hoeveel-mensen-hebben-overgewicht.

[18] Nederkoorn C., Houben K., Hofmann W., Roefs A., Jansen A. (2010). Control yourself or just eat what you like? Weight gain over a year is predicted by an interactive effect of response inhibition and implicit preference for snack foods. Health psychology : official journal of the Division of Health Psychology, American Psychological Association.

[19] Netherlands Nutrition Centre (2011). Richtlijnen voedingskeuze.

[20] Netherlands Nutrition Centre (2013). Caloriechecker.

[21] O’Connor D. B., Jones F., Conner M., McMillan B., Ferguson E. (2008). Effects of daily hassles and eating style on eating behavior. Health Psychology.

[22] Piernas C., Popkin B. M. (2010). Snacking increased among U.S. Adults between 1977 and 2006. Journal of Nutrition.

[23] Reis H. T., Gable S. L., Maniaci M. R., Reis H. T., Judd C. M. (2014). Methods of Studying everyday experience in its natural Context. Handbook of Research Methods in Social and Personality Psychology.

[24] Rijksinstituut Volksgezondheid en Milieu (2011). NEVO-online version 2011/3.0.

[25] Robinson M. D., Clore G. L. (2002). Belief and feeling: Evidence for an accessibility model of emotional self-report. Psychological Bulletin.

[26] Schwarz N., Kahneman D., Xu J., Belli R., Stafford F. P., Alwin D. F. (2009). Global and episodic reports of hedonic experience. Calendar and time diary methods in life course research.

[27] Scollon C. N., Kim-Pietro C., Diener E. (2003). Experience sampling: Promises and pitfalls, strengths and weaknesses. Journal of Happiness Studies.

[28] Silvia P. J., Kwapil T. R., Eddington K. M., Brown L. H. (2013). Missed beeps and missing data: dispositional and situational predictors of nonresponse in experience sampling research. Social Science Computer Rev.

[29] StataCorp (2009). Stata Statistical Software: Release 11.

[30] Stephen A. M. (2007). The case for diet diaries in longitudinal studies. International Journal of Social Research Methodology.

[31] Stone A. A., Shiffman S. (1994). Ecological momentary assessment (EMA) in behavioral medicine. Annals of Behavioral Medicine.

[32] Strien T., van Frijters J. E. R., Bergers G. P. A., Defares P. B. (1986). The Dutch Eating Behavior Questionnaire (DEBQ) for assessment of restrained, emotional and external eating. International Journal of eating disorders.

[33] Thewissen V., Bentall R. P., Oorschot M., Campo J. à., van Lierop T., van Os J., Myin-Germeys I. (2011). Emotions, self-esteem, and paranoid episodes: An experience sampling study. British Journal of Clinical Psychology.

[34] Thompson F. E., Subar A. F., Coulston A. M., Boushey C. J. (2008). Dietary assessment methodology. Nutrition in the prevention and treatment of disease.

[35] Thompson F. E., Subar A. F., Loria C. M., Reedy J. L., Baranowski T. (2010). Need for technological innovation in dietary assessment. Journal of the American Dietetic Association.

[36] Tournier M., Sorbara F., Gindre C., Swendsen J. D., Verdoux H. (2003). Cannabis use and anxiety in daily life: A naturalistic investigation in a non-clinical population. Psychiatry Research.

[37] Tulving E. (1984). Précis of elements of episodic memory. The Behavioral and Brain Sciences.

[38] Wouters S., Jacobs N., Duif M., Lechner L., Thewissen V. Affect and between-meal snacking in daily life: the moderating role of gender and age.

[39] Wouters S., Thewissen V., Zamani K., Lechner L., Jacobs N. (2013). Snackimpuls: een smartphone applicatie gericht op de onbewuste determinanten van snackgedrag. De Psycholoog.

